# On demand delivery and analysis of single molecules on a programmable nanopore-optofluidic device

**DOI:** 10.1038/s41467-019-11723-7

**Published:** 2019-08-16

**Authors:** M. Rahman, M. A. Stott, M. Harrington, Y. Li, M. J. N. Sampad, L. Lancaster, T. D. Yuzvinsky, H. F. Noller, A. R. Hawkins, H. Schmidt

**Affiliations:** 1 School of Engineering, University of California Santa Cruz, 1156 High Street, Santa Cruz, CA 95064 USA; 2 ECEn Department, Brigham Young University, 459 Clyde Building, Provo, UT 84602 USA; 3 Department of Molecular, Cell and Developmental Biology and Center for Molecular Biology of RNA, University of California at Santa Cruz, 1156 High Street, Santa Cruz, CA 95064 USA

**Keywords:** Nanoscience and technology, Engineering

## Abstract

Nanopore-based single nanoparticle detection has recently emerged as a vibrant research field with numerous high-impact applications. Here, we introduce a programmable optofluidic chip for nanopore-based particle analysis: feedback-controlled selective delivery of a desired number of biomolecules and integration of optical detection techniques on nanopore-selected particles. We demonstrate the feedback-controlled introduction of individual biomolecules, including 70S ribosomes, DNAs and proteins into a fluidic channel where the voltage across the nanopore is turned off after a user-defined number of single molecular insertions. Delivery rates of hundreds/min with programmable off-times of the pore are demonstrated using individual 70S ribosomes. We then use real-time analysis of the translocation signal for selective voltage gating of specific particles from a mixture, enabling selection of DNAs from a DNA-ribosome mixture. Furthermore, we report optical detection of nanopore-selected DNA molecules. These capabilities point the way towards a powerful research tool for high-throughput single-molecule analysis on a chip.

## Abstract (web summary)

Nanopore based detection has numerous high impact applications outside of next-generation sequencing. Here the authors use an optofluidic chip to detect individual individual 70S ribosomes in a sample mixed with DNA.

## Introduction

In recent years, single molecule detection and analysis (SMA) has rapidly grown into an important and vibrant field and is employed across a wide range of disciplines such as molecular biology, analytical chemistry, biomedicine, biophysics, physiology, genomics, and proteomics^1,2^. SMA of nucleic acids, proteins, ribosomes and other biomolecules has enabled new discoveries in biological processes and a deep understanding of cellular function and therapies based on molecular functions^3–5^. Optical methods such as fluorescence and Raman microscopy have played an important role in the development of the field^6,7^. More recently, two new fields with direct impact on single biomolecule analysis have emerged. One is electrical single-molecule analysis using nanopores in which translocations of individual particles through a nanoscopic opening in a membrane cause characteristic changes in ionic current flowing through the membrane^8–12^. Depending on the salt concentration and the chemical properties of the pore, the current modulation manifests as a decrease or increase in the ionic current^13,14^. In this manner, nanopore detection forms the basis for low-cost, next-generation sequencing applications^8–10^, but is also increasingly being considered for other molecular targets such as proteins or metabolites^11,12^. The second development is the emergence of optofluidic devices that combine microfluidics with integrated optics in a single system^15,16^. Various implementations of this concept have resulted in demonstrated optical sensitivity to single biomolecules such as viruses^17^ and nucleic acids^18^. These two technologies have also been combined by successful integration of nanopores with liquid-core waveguide-based optofluidic chips to simultaneously implement electrical and optical single nanoparticle sensitivity^19,20^.

An ideal SMA research tool would allow for selection of a desired target molecule from a potentially complex mixture for subsequent analysis. Nanopores can be a key component of such a system if they can be operated as gates that enable transfer of a desired number of target molecules from a reservoir to a separate analysis region. Indeed, several efforts to implement rudimentary nanopore gating have already been made such as recapturing molecules after translocation^21,22^, turning on/off a nanopore by wetting-dewetting the pore^23–25^, and the use of a pulsed DC source to turn on/off the pore with an adjustable sub-Hz frequency^26,27^. However, controlled particle delivery based on real-time translocation detection on a full chip-scale platform has remained elusive.

Here, we show that nanopore-optofluidic devices can provide all these functionalities on a single chip. By integrating a feedback control system with the nanopore-optofluidic chip, it is possible to achieve precise control over particle delivery into the microfluidic channel. The adjustable settings of the feedback control system allow the user to reconfigure the platform depending on the application. Specifically, we show that feedback-controlled nanopore gating can be applied to a wide range of biomolecules, including controlled introduction of a user-defined number of molecules and selective gating of a specific molecular target from a mixture of molecules passing through the pore. This enables controlled delivery of individual molecules for further optical analysis, here fluorescence detection of gated DNA molecules, at user-programmable rates up to the kHz range.

## Results

### Chip design and experimental setup

**Fig. 1:**
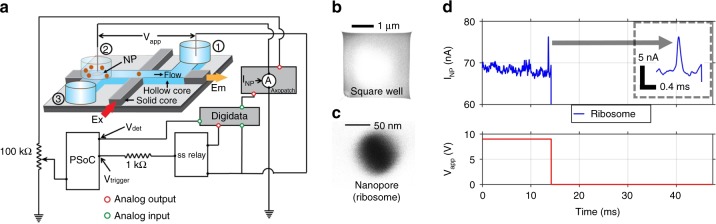
On-demand single molecule analysis platform. **a** A nanopore-optofluidic chip combines liquid-core (blue) and solid-core (gray) optical waveguides; particles are introduced into the liquid-core waveguide by applying a voltage across reservoirs 1 and 2, where reservoir 2 is placed over a nanopore; fluorescence is excited via an intersecting solid-core waveguide and collected by the liquid-core waveguide. The chip is connected to an electronic circuit for implementing feedback control over the number of particles entering the channel through the nanopore **b** SEM image of a typical square well milled on the microchannel to remove the thick oxide layer for further nanopore drilling. **c** SEM image of a drilled nanopore. **d** Experimental demonstration of voltage-gated delivery of a single 70 S ribosome into the microfluidic channel; top: nanopore current (inset: zoomed in translocation), bottom: voltage applied across the nanopore

The chip-based single-molecule analysis system is based on a nanopore-optofluidic chip integrated with a feedback-control circuit as illustrated in Fig. 1a. Details of the chip composition and fabrication are provided in the Methods section. Briefly, a microfluidic channel (blue) that acts as a liquid-core optical waveguide^17,18^ is connected to conventional solid-core waveguides (gray) that enable planar, fiber-optic access to and from the chip. The channel ends are terminated by attached fluidic reservoirs for introduction of sample materials and application of electrical and/or mechanical driving forces. In addition, a third reservoir is placed over a nanopore that has been ion-milled into the layer covering the fluidic waveguide channel^19,20,28^. SEM images after a typical ion milling process are shown in Fig. 1b, c. A voltage applied between reservoirs 1 and 2 pulls single analyte particles into the fluidic channel.

### Programmable nanopore gating of single biomolecules

The first core element of the single-molecule analysis platform is the introduction of feedback control over particle delivery into the channel. This was implemented by adding a microcontroller and a solid-state relay as schematically shown in Fig. 1a. The microcontroller carries out real-time analysis of the current through the nanopore. Whenever a current modulation exceeds a predetermined threshold value, translocation of a single particle is registered while random baseline fluctuations are filtered out. The solid-state relay is used to shut off the nanopore driving voltage after a user-defined number of particles have moved through the nanopore. Additional details about the control circuit and how it is used to identify particle translocation events are provided in the Methods section.

This feedback control system offers deterministic delivery of single bio-molecules into the analysis region. Figure 1d shows the controlled introduction of a single 70S ribosome through a 38 nm wide nanopore into the microfluidic channel. The top part of Fig. 1d shows the current trace, and the bottom part shows the corresponding trace of the voltage applied across the pore. The voltage across the nanopore was turned off after the current had returned to its baseline after the single-particle translocation. The nanopore dimensions relative to the size of the 70S ribosome permit only one ribosome at a time in the pore so that the voltage gating ensured capture of a single target particle inside the analysis region by preventing further molecular insertion.

**Fig. 2:**
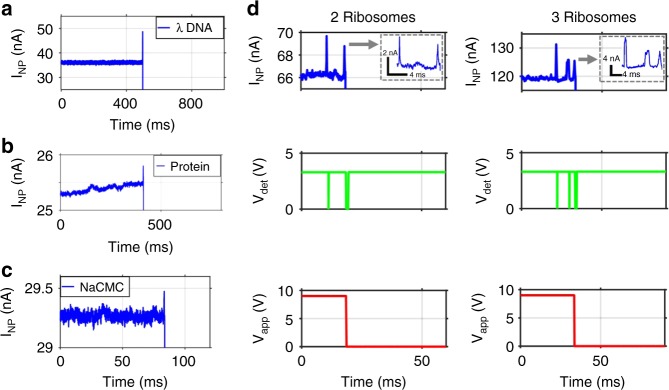
Gated delivery of single bioparticles with reconfigurable settings. **a**–**c** Current trace of voltage-gated delivery of single λ-DNA, Zika NS-1 protein, and NaCMC molecule respectively, into the microfluidic channel. **d** Example of delivery of user-defined particle number (here: two and three) into fluidic channel (top: current through pore (inset: zoomed in translocations); center: signals in detection circuit of digitized identification of particle translocation; bottom: voltage across pore turned off after the desired number of particles has been detected

This ability to control particle delivery via a nanopore is further enhanced by the ability to control key aspects of the feedback process. First, we demonstrate that this process works for a diverse range of target particles and fluidic environments. Figure 2a–c show gated translocations of single λ-DNAs, Zika virus NS-1 proteins, and sodium carboxymethyl cellulose (NaCMC) molecules, respectively. These molecules were detected from a different buffer solution through a smaller (20 nm wide) pore, validating the broad applicability of our feedback control system for vastly different target molecules and nanopore dimensions. We note that, while most of the measured λ-DNA translocations were from individual molecules, we also observed multi-peak translocations. These are due to the fact that the nanopore was much wider than the λ-DNA molecules, allowing for multiple targets to pass through the pore nearly simultaneously. This can be prevented by using a much narrower pore or by discarding multi-particle events.

**Fig. 3:**
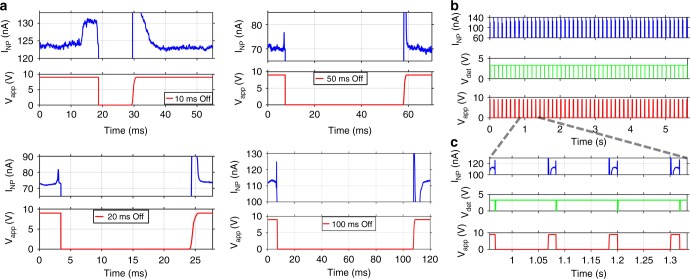
Automated delivery of successive 70S ribosomes. **a** Automatic re-application of voltage across the pore after user-defined time delays (here: 10, 20, 50, and 100 ms) during which the pore is closed. Note that the vertical axis limit for I_NP_ has been chosen for best visibility of the molecular signatures. The saturation limit of the setup is 200 nA. **b** Demonstration of automated delivery of 48 ribosomes (~513/min) into the microfluidic channel where the voltage is re-applied 100 ms after each translocation; top: nanopore current, center: translocation detection pulses, bottom: voltage applied across pore; **c** zoomed-in view of panel **b** revealing translocation events and how pore is switched off for 100 ms after a translocation has been detected

Another important feature of our feedback control system is that important experimental parameters are user-programmable. First, we show that the control algorithm can be programmed to translocate a desired number of particles before the pore is turned off. Figure 2d illustrates this capability for the deliberate delivery of two and three 70S ribosomes into the optofluidic channel, respectively. A second implementation of this user-defined control is automatic re-opening of the nanopore gate after a desired time interval following a translocation event. In other words, the pore can be kept in its closed state for a user-defined amount of time before it is re-opened. Figure 3a shows examples of this feedback-controlled delivery for four different durations of the closed-state. Once the analysis time of a single molecule is known or predicted, the closed time can be set to a desired duration for successive single molecule delivery in an automated fashion.

Finally, the system can be used for rapid, highly controlled delivery of single molecules into the channel at high rates. Figure 3b illustrates this automated single molecule delivery by showing 48 gated translocation events of individual 70S ribosomes (top), corresponding detection pulses produced by the control circuit (center), and the voltage applied across the pore (bottom). This measurement represents gated particle introduction into the fluidic channel at a rate of >500/min. Figure 3c illustrates a closeup view of a small time segment which reveals that each of the signals in Fig. 3b is a highly controlled translocation event of a single ribosome as shown in Fig. 1d. Every time a translocation was identified in the current signal, the voltage across the pore was turned off, preventing translocation of another ribosome and the pore was automatically re-opened 100 ms after each translocation. In the present configuration, the feedback control circuit is limited by the rise time to reopen the pore of ~1.5 ms (Fig. 3a) and can rapidly deliver single particles up to rates of ~625 Hz. We note that the nanopore current signals in Fig. 3 show capacitively induced transients when the pore is reopened. Detection of translocations and activation of gating is still possible during these transients. This would not be possible if the current is saturated at its present limit of 200 nA which occurred only in the case of the first signal shown in Fig. 3a. Even there, the saturation dead time of 1.0 ms was significantly shorter than the voltage rise time (1.5 ms), which means that the applied voltage during the dead time was still too low to produce spurious translocations.

### Selection of single biomolecules from a mixture

**Fig. 4:**
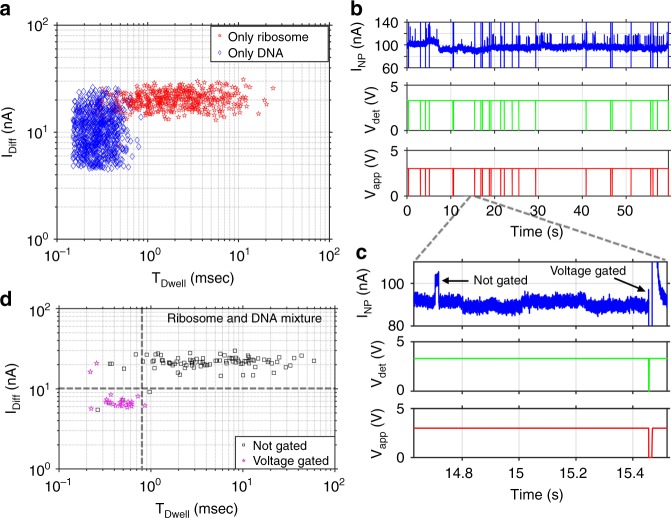
Voltage-gated selection of specific particles from a mixture. **a** Scatter plot of differential current vs. dwell time of translocation events when only λ-DNAs (blue) and only ribosomes (red) are drawn through the same nanopore. **b** Demonstration of selective voltage gating of λ-DNAs from a mixture of ribosome and λ-DNA; top: current through nanopore, center: specific translocation detection pulses, bottom: voltage applied across pore; **c** zoomed-in view of panel **b**, revealing how specific targets are voltage-gated while others are not gated. **d** Scatter plot of differential current vs. dwell time of each translocation event of panel **b**, where magenta points show voltage-gated translocations, and black points show the translocations which are not gated, with thresholds shown by dotted lines

An important feature of a nanopore-based molecular analysis system is the ability to identify and select a particular molecule from a mixture. We demonstrate this capability by selectively gating λ-DNA mixed with 70S ribosomes, i.e., to activate the gating function and close the pore only if DNA has entered the channel. In other words, the DNA gating process is robust to contamination by other particles with different translocation signatures. This can enable, for example, fluorescence experiments on a controlled number of DNAs while unlabeled particles (here: ribosomes) are ignored and discarded. Now, the circuit has to not only respond to the leading edges of a translocation; rather, it must analyze the full translocation (amplitude, duration) pattern in real time and respond rapidly enough for selective gating. Scatter plots of dwell time vs. differential current of each translocation event of the control experiments are shown in Fig. 4a where only ribosomes (red) and only λ-DNAs (blue) move through the nanopore, respectively. Figure 4a shows that translocations with differential amplitude less than 10 nA and dwell time less than 0.8 ms are generated by λ-DNAs, and these values are used as criteria for selective gating of DNAs into the fluidic channel. Note that a lower boundary for the differential amplitude of 4.5 nA was selected as the average background current plus three times the standard deviation of the current noise. As the loop run time of the microcontroller is known (~0.104 ms for 5 samples/iteration), the dwell time of translocations can be determined and, by observing peak height, the amplitude of translocations can also be tracked. This tracking ability is used to detect translocations specific to λ-DNAs from a mixture of ribosomes and λ-DNAs simultaneously going through the same nanopore. The top trace in Fig. 4b shows variation of the current through the nanopore with translocations produced by the mixture of ribosomes and λ-DNAs. The center trace shows detection signals for translocations which fulfill the amplitude and duration criteria and the bottom trace shows how the voltage across the pore was turned off for 10 ms after each specific translocation. Figure 4c depicts a zoomed-in view of a small time segment of Fig. 4b, which reveals how specific translocations are voltage-gated based on predetermined criteria. The scatter plot of dwell time vs. differential current for all translocation events is shown in Fig. 4d with voltage-gated (magenta) and not voltage-gated (black) events. The dotted lines illustrate the amplitude (~10 nA) and duration threshold (~0.8 ms) applied for selective voltage gating. In order to determine the accuracy of this process, we consider the 119 translocation events represented in the trace shown in Fig. 4b. Among them, 24 are voltage gated, 3 are missing and 3 are incorrectly gated. This yields a total of 21 true positives (TP) and 92 true negatives (TN). The accuracy is calculated using

1 $${\mathrm{Accuracy}} = \frac{{{\mathrm{TP}} + {\mathrm{TN}}}}{{{\mathrm{Total}}}} = \frac{{21 + 92}}{{119}} = 94.96{\mathrm{\% }}$$

This shows that high accuracy levels can be achieved with our feedback control system. We note that the microcontroller can be reprogrammed to voltage-gate ribosomes by selecting events with long dwell times and large differential current changes if desired.

### Optical detection of gated molecules

**Fig. 5:**
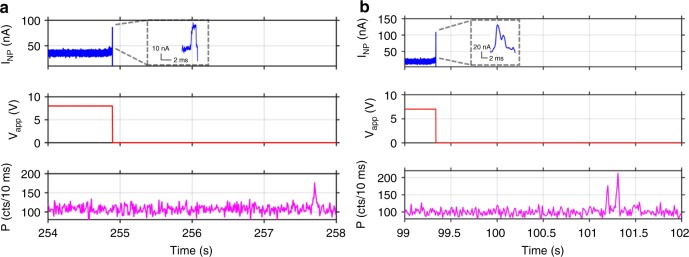
Fluorescence detection of λ-DNA molecules on demand. **a** top: Current through pore and electrical detection of a single λ-DNA entering fluidic channel (dashed box: zoomed-in view of translocation event); center: voltage across the pore preventing further translocations after event has been detected; bottom: Concurrently recorded fluorescence signal showing optical detection after characteristic transport time. **b** Introduction and detection of two λ-DNA molecules; the ambiguity of the double-peak electrical signal is removed by the optical detection after both DNA molecules have separated in the channel

The analysis system can also be used for optical detection of the bio-molecules/nanoparticles as reported previously^19,20^, but now for a user-defined number of molecules. Specifically, the optofluidic chip provides sufficient sensitivity for detecting individual labeled nucleic acids^18,20^. In Fig. 5a, we show how one single λ-DNA molecule was introduced into the chip (top panel), how the nanopore was closed by turning off the applied voltage (center panel), and how the molecule was subsequently detected optically (bottom panel), demonstrating on-demand fluorescence detection of one, and only one, molecule on a chip. We can use the presence of both electrical and optical signals to calculate the flow velocity of the DNA in the microfluidic channel. By dividing the physical spacing between nanopore and waveguide intersection by the observed time difference, we find a velocity of 33.9 μm/s.

The dual-mode electro-optical detection capability also offers a key advantage for ensuring single particle screening in the case where near-simultaneous translocations from multiple molecules can create ambiguous current blockades. This case is illustrated in Fig. 5b, where the top and center panels show how the pore was closed after detection of a double-peak translocation signal. Given the relatively large size of the nanopore relative to the DNA molecule, it can be expected that some translocation signals consist of multiple particles moving through the pore at the same time. However, such multi-peak signals could also arise from non-standard translocations of single molecules, e.g., folded DNA. From the electrical signal alone, it is impossible to identify the nature of the event. However, the optical trace (bottom panel) clearly reveals that two DNA molecules translocated through the pore and had separated sufficiently by diffusion when they reached the optical interrogation spot. Note that in an application demanding true single-molecule delivery, the particles could easily be discarded based on this optical signal, and the pore can be reopened until a single target has indeed been delivered.

## Discussion

In summary, we have introduced a single-molecule analysis platform for controlled single-molecule delivery and analysis. Automated introduction and detection of single 70S ribosomes was demonstrated. Identification and voltage gating of specific target molecules from a mixture based on individual molecular translocation pattern was implemented. This platform can be used to control the number of a specific particle type for further measurements or for down-stream fluidic sorting or purification of different molecules after identification. Furthermore, the system allows user flexibility with reconfigurable settings depending on application requirement and necessity. The combination of feedback-controlled introduction of single molecules into a fluidic channel through a nanopore with subsequent fluorescence detection enables the optical analysis of many single molecules in rapid succession, pointing the way towards on-demand high-throughput single-molecule analysis on a chip. In the future, this feedback control system can also be integrated with anti-Brownian electro-optical (ABEL) trapping, which has been shown to be effective down to the size of single dye molecules^29^. Integration of this trapping concept in one and two dimensions using optofluidic waveguides has shown excellent promise and points towards on-chip prolonged analysis of single nanopore selected molecules^30,31^.

## Methods

### Nanopore-optofluidic chip fabrication

Optofluidic chips were created on top of a <100> oriented Si substrate. Six alternating dielectric layers of SiO_2_ (*n* = 1.47) and Ta_2_O_5_ (*n* = 2.107) were then sputtered over the whole wafer to thicknesses of 265 nm and 102 nm respectively, forming the ARROW layer stack, which acts as the substrate in subsequent fabrication steps. The wafers were oriented so that chip edges aligned with <110> cleavage planes to produce clean optical faces when the wafer was cleaved into individual chips approximately 1 cm^2^ in size. The 6 × 12 µm hollow-core microchannels, which carry fluids and micro/nano-particles, were defined using standard lithography procedures for SU-8, and then hard baked at a temperature of 250 °C. A self-aligned pedestal was defined by reactive ion etching (RIE) to improve the optical throughput and structural integrity of the hollow cores. Once the pedestal was defined, a 6 µm thick, low-stress PECVD oxide layer was deposited on top. Collection waveguides were then patterned by photolithography and etched by RIE to create 5 µm tall rib waveguides. The SU-8 cores were exposed at the ends by removing the oxide with buffered hydrofluoric acid and then placed in a strong acid to remove the SU-8, hollowing out the liquid-core channel.

An FEI Quanta 3D FEG DualBeam SEM/FIB was used to fabricate nanopores on the hollow core channel. For each nanopore, a square well was first milled most of the way through the ~6 μm thick top oxide layer using a focused Gallium ion beam (FIB) operating at 30 kV, 0.5–1 nA, leaving an intact thin membrane on top of the hollow channel as shown in Fig. 1b. Nanopores were milled into the membrane by exposure to a 1.6–10 pA, 30 kV Ga beam for suitable time periods, with exposure controlled by the Nanometer Pattern Generation System (NPGS, JC Nabity).

### Nanopore feedback control circuit

A Digidata 1440 A digitizer (Molecular Devices) was used to generate and record the voltage and current through the nanopore. A patch-clamp amplifier (Axopatch 200B, Molecular Devices) was used to measure the current through the nanopore (I_NP_), with a low- pass filter tuned to a cutoff frequency of 10 kHz. Standard Ag/AgCl electrodes were used for the experiments. To implement the feedback control gating, a microcontroller (PSoC 5lp, Cypress Semiconductor) was used. The output signal of the Axopatch (V_INP_) was sent to the analog to digital converter (ADC). The signal was fed through a potentiometer (100 kΩ) to fine-tune the voltage level for the ADC. A user defined voltage generated by using the Digidata was sent to the input terminal of a solid-state relay (Vishay Semiconductors). The microcontroller outputs a logical 1 voltage signal (V_trigger_) to the relay control terminal, causing the relay to apply the Digidata voltage across reservoirs 1 and 2 (V_app_). As soon as the microcontroller detects the desired number of translocations, it sends a logical 0 (zero voltage) signal to the relay terminal and the relay disconnects the circuit, resulting in zero applied voltage across the reservoirs, prohibiting any further particle translocations until the voltage is reset. When the microcontroller detects a translocation, it also generates a pulse voltage (V_det_) as a detection signature, which is sent to the Digidata for recording the number of translocation events.

In order to distinguish a particle translocation event from other current variations (noise, drift), the microcontroller calculates the standard deviation (SD) from a certain number of initial samples and defines a threshold, which varies depending on the target. A reference is initialized by calculating the mean of the samples. After the initialization stage, a rolling average is calculated from a fixed number of samples (5 in this case) on a continuous basis and each average point is compared with the reference to monitor whether it exceeds the threshold. In every cycle, the reference is also updated unless the average point exceeds the threshold. As soon as an average point exceeds the threshold, it is considered as the start of a translocation. Next, the peak height is calculated and the ongoing average is monitored to determine whether it falls back to a desired value surrounding the baseline within a characteristic time period (the maximum predicted duration of a translocation; varies depending on the target). If the average does not fall below the predetermined value within the desired time it is discarded as baseline noise, the variables are reset, and the process continues. Additional signal processing is implemented to identify translocations more accurately. Only the pulses which exceed the threshold and fall back within the characteristic time are considered and detected as translocations.

### Optical setup

For optical detection of λ-DNA, a 488 nm wavelength laser (White light source, NKT Photonics) was used. The fiber coupled laser light was end-to-end coupled into the excitation solid core (SC) waveguide. The in-plane signal was collected at the orthogonal collection solid-core waveguide facet using a butt-coupled multimode fiber. The spectrally filtered fluorescence signal was sent to a single photon avalanche photodiode (Excelitas) via a connectorized multimode fiber.

### Sample preparation

At the beginning of each solution preparation step, target-specific buffers were filtered with 20 nm Whatman Anotop syringe filters (GE Healthcare) to remove any unwanted contamination.

Ribosomes were purified from E. coli MRE600 cells grown at 37 °C to mid-log. Cells were lysed through a French press at 18,000 psi. The lysate was clarified by centrifugation at 30,000 × *g* for 30 min in a JA20 rotor (Beckman) before layering onto cushions containing 1.1 M sucrose, 10 mM Tris-HCl (pH 7.5), 500 mM NH_4_Cl, 10 MgCl_2_, and 6 mM βME, and ultracentrifuged in a Beckman Ti70 rotor for 20.5 h at 106,000 × *g*. The resulting ribosome pellet was resuspended in 20 mM Tris-HCl (pH 7.5), 500 mM NH4Cl, 10 mM MgCl_2_, and 6 mM BME and re-pelleted twice at 223,000 × *g* for 2 h in a Ti70 rotor (Beckman). The resuspended pellet was then loaded onto 10-35% sucrose gradients containing 20 mM Tris-Cl (pH 7.5), 100 mM NH4Cl, 6 mM MgCl2, and 6 mM βME, and ultracentrifuged in a Beckman SW28 rotor at 48,000 × *g* for 17 h. The 70 S ribosome peak was collected from the gradients using a BioComp Piston Gradient Fractionator, then ultracentrifuged in a Ti45 rotor (Beckman) at 101,000 × *g* for 22 h. The ribosome pellet was resuspended in 50 mM KHepes (pH 7.9), 100 mM KCl, 10 mM MgCl_2_, and 6 mM βME. Aliquots were flash-frozen in liquid N_2_ and stored at −80 °C.

λ-DNAs (New England Lab) were diluted into the 1× T50 buffer (10 mM Tris-HCl, 50 mM NaCl) to a final concentration of 9.55 × 10^11^/mL. For optical detection of λ-DNA, 45 µL of 9.55 × 10^11^/mL λ-DNA aliquot (suspended in 1× T50) was mixed with 5 µL of 2× SYBR Gold (Invitrogen) intercalating dye.

Zika virus nonstructural 1 (NS-1) proteins (EastCoast Bio) were diluted in 1xT50 buffer from stock concentration to a final concentration of 5.12 × 10^12^/mL.

NaCMC solution was prepared by dissolving 26 mg of NaCMC powder (Sigma-Aldrich, Product Number: 419273) into 1 mL of 1× T50 buffer.

### Reporting summary

Further information on research design is available in the Nature Research Reporting Summary linked to this article.

## Supplementary information

Reporting Summary

## Data Availability

The data that support the findings of this study are contained within the manuscript and its supporting documents and are available from the corresponding author upon reasonable request.

## Code availability

The code used for data analysis is available from the corresponding author upon reasonable request.
